# Role of Lymphadenectomy in the Management of Early-Stage Endometrial Cancer

**DOI:** 10.7759/cureus.82408

**Published:** 2025-04-16

**Authors:** Kheyal Azam Khalil, Maria Habib, Sana Hussain, Muhammad Usman, Aamir Ali Syed

**Affiliations:** 1 Surgical Oncology, Shaukat Khanum Memorial Cancer Hospital and Research Centre, Lahore, PAK

**Keywords:** adjuvant chemotherapy, disease-free survival, endometrial neoplasms, lymph node excision, sentinel lymph node

## Abstract

Objective

This study aimed to determine the role of pelvic lymphadenectomy by assessing nodal positivity on progression-free and overall survival in early-stage endometrial cancer.

Materials and methods

Eighty-nine women diagnosed with stage I/II endometrial cancer at presentation who underwent pelvic lymphadenectomy during surgery from 2019 to 2023 were included in this retrospective study. Data was collected using the Hospital Information System (HIS), and patient identifiers were anonymized. In addition to patient characteristics, final histopathology including cytology, type of surgery (laparoscopic vs. open), radiological evidence of lymphadenopathy before surgery, number of lymph nodes retrieved, histopathological evidence of nodal-positive/nodal-negative disease, adjuvant therapy (if any), recurrence-free survival, and overall survival were noted for these patients. Analysis was done using IBM SPSS Statistics for Windows, Version 25.0 (Released 2017; IBM Corp., Armonk, New York, United States), with means and frequencies noted for descriptive variables. Recurrence-free survival and overall survival were estimated in months for patients with lymph node-positive and lymph node-negative disease.

Results

Fifty-eight (65.2%) patients underwent laparoscopic surgery in this study cohort with radiological evidence of lymphadenopathy in 17 patients. Only six patients were found to have nodal-positive disease, out of which only three had lymphadenopathy on scans. Forty-six (51.7%) patients received adjuvant radiation therapy, while 16 (18%) underwent adjuvant chemotherapy. The estimated mean survival was 65.6 months, with the recurrence-free survival being 61.7 months. Among the patients with lymph node-positive disease, only one was found to have disease recurrence despite adjuvant treatment.

Conclusion

This study elucidates that patients who underwent pelvic lymphadenectomy and were subsequently found to have nodal disease went on to receive adequate adjuvant therapy; however, for some of them, there was no specific preoperative indicator to prompt the decision for pelvic lymphadenectomy. Therefore, until advanced techniques such as sentinel lymph node mapping are available in low-resource countries, surgical staging with pelvic nodal sampling is recommended.

## Introduction

Endometrial cancer is one of the commonly occurring gynecological cancers presenting at our institution as well as worldwide [1]. Surgical intervention is an integral part of the management of endometrial cancer [2]; surgical staging has been around for over 20 years since the Gynecologic Oncology Group (GOG) prospective trial in 1984 [3]. 

A change in perspective was required, however, when the role of the extent of surgery was looked into for early-stage endometrial cancer that posed an important question: assessing the need for pelvic lymphadenectomy and its safety and efficacy versus the long-term sequelae of lymphadenectomy [4]. The advent of adjuvant therapy also augmented this lens shift, as determining the need and type of adjuvant therapy in early-stage endometrial cancer was also reliant on the histopathologic spread of disease established post-surgery [5]. Since then, local as well as international guidelines have been conflicting [6]. There is a school of thought that systemic lymphadenopathy may pose an unacceptable rate of morbidity that might not offer significant survival benefit; therefore, every effort should be made to minimize the radicality of surgery when possible. Lately, sentinel lymph node mapping is becoming the standard of care for early-stage endometrial cancer to guide pelvic lymph node dissection [7]. 

Low-resource centers are still reliant on pelvic lymphadenectomy for staging early endometrial disease. With the advent of molecular classification and risk stratification of endometrial cancer, in combination with sentinel lymph node mapping, many centers may be able to minimize, if not eliminate, the need for systemic lymphadenectomy. However, with a paucity of resources for these advanced preoperative tools, many centers rely on lymph node dissection for surgical staging. 

In the face of limitations of radiological staging [8] and possibility of change in histopathological diagnosis of the final specimen compared to endometrial biopsy diagnosis, this study aims to determine, in our institution, the role of pelvic lymphadenopathy, the rate of nodal positive disease and its associated histopathology, and the comparison of progression-free survival and overall survival in patient who had nodal-positive versus nodal-negative disease in stage I/II endometrial cancer. The results will help determine if the current practices regarding pelvic lymphadenectomy during surgery for early-stage endometrial cancer are truly offering a survival benefit and may prompt the need for further investigation regarding choosing patients who warrant a pelvic lymphadenectomy. With the awareness of the gradual decline of systemic lymphadenectomy worldwide, we want to evaluate and analyze what we can do differently with the information and tools that we have access to to determine the indication of lymphadenectomy. Our patient population will benefit from this while minimizing the morbidity associated with extensive surgery. 

## Materials and methods

Two hundred and seventy-two women diagnosed with stage I/II endometrial cancer at presentation who underwent surgery were obtained from the cancer registry of Punjab. Eighty-nine of these were found to fulfill the inclusion criteria of this study which was defined as women with early-stage endometrial cancer who underwent pelvic lymphadenectomy during surgery from 2019 to 2023. 

Exclusion criteria were as follows: women with endometrial cancer who did not undergo pelvic lymphadenectomy and/or with no histopathological evidence of lymph node tissue on the submitted specimen, women with advanced stage III/IV endometrial cancer on presentation, those with an Eastern Cooperative Oncology Group (ECOG) performance status of >3 at presentation, those with a body mass index (BMI) of >40 kg/m^2^, women who were lost to follow up, have more than one primary cancer, and with incomplete adjuvant therapy data, and patients who contracted COVID-19 during treatment. 

It is pertinent to note here that patients who did not undergo pelvic lymph node dissection were not included in this study for comparison as has been the case in previous similar studies due to a number of reasons, the most important one being that patients who had a clear indication for lymph node dissection such as radiological evidence of lymphadenopathy or intraoperative finding of enlarged lymph nodes underwent lymph node dissection irrespective of initial stage determined at presentation. Therefore, the women undergoing pelvic lymph node dissection in early-stage endometrial cancer in our study cohort were inherently not comparable to those who did not have pelvic lymph node dissection, and the comparison would be biased when considering progression-free survival and overall survival. 

Data was collected using the Hospital Information System (HIS), and patient identifiers were anonymized. Approval from the Institutional Review Board of Shaukat Khanum Memorial Cancer Hospital and Research Centre was obtained prior to data collection for this retrospective study (approval number: EX-01-02-24-02). Non-probability consecutive sampling technique was used. In addition to patient characteristics such as age and BMI, final histopathology including cytology, type of surgery (laparoscopic vs. open), radiological evidence of lymphadenopathy before surgery, number of lymph nodes retrieved, histopathological evidence of nodal-positive/nodal-negative disease, adjuvant therapy (if any), recurrence-free survival, and overall survival were noted for these patients (in case of death of the patient during treatment or at follow-up). Analysis was done using IBM SPSS Statistics for Windows, Version 25.0 (Released 2017; IBM Corp., Armonk, New York, United States), with means and frequencies noted for descriptive variables. Recurrence-free survival and overall survival were estimated in months for patients with lymph node-positive and lymph node-negative disease. 

## Results

All 89 patients were diagnosed with endometrial cancer based on endometrial biopsy. Radiological evaluation was then performed to predict the stage of the disease. All patients were deemed in the early stage (stage I/II) based on clinical and radiological information preoperatively and decided for upfront surgical staging surgery after a multidisciplinary team meeting. The mean age of this study cohort was 54.8 years (range: 32-80 years). The BMI reported was 32.2 kg/m^2 ^with a standard deviation of 6.2. Out of these 89 patients, 58 underwent surgery with a laparoscopic approach, while 31 had open surgery with a midline incision. The details of patients with radiological evidence of lymphadenopathy, adjuvant chemotherapy, adjuvant radiation, and lymph node status on histopathological diagnosis are mentioned below. Only two patients underwent para-aortic lymphadenectomy in addition to pelvic lymph node dissection; routine systemic lymphadenectomy included bilateral pelvic lymph node dissection. The mean number of lymph nodes retrieved was 7.72 (a minimum of 1 and a maximum of 31 lymph nodes were removed). Seventeen patients had lymphadenopathy on radiology scans. Six patients were found to have nodal-positive disease, out of which only three had lymphadenopathy on scans.

Table 1 lists the histopathological types of endometrial cancer, with endometrioid adenocarcinoma being the most common histology.

**Table 1:** Histopathological types of endometrial cancer

Histology	Number of patients
Endometrioid adenocarcinoma	64
Endometrial stromal sarcoma	4
Poorly differentiated/dedifferentiated adenocarcinoma	3
Leiomyosarcoma	2
High-grade serous carcinoma	7
Clear cell carcinoma	3
Mixed endometrioid and clear cell carcinoma	1
Mixed endometrioid and serous carcinoma	1
Carcinosarcoma	1
Adenosarcoma	1

Twenty-six patients had cytological evaluation of peritoneal fluid, at the discretion of the operating surgeon, out of which four were positive for malignant cells. Forty-six (51.7%) patients received adjuvant radiation therapy, while 16 (18%) underwent adjuvant chemotherapy. Adjuvant chemotherapy regimens included carboplatin/paclitaxel for primary disease and gemcitabine, cisplatin, or doxorubicin for recurrent disease. Twenty-three patients received vaginal brachytherapy (BT), and seven received extended beam radiation therapy (EBRT), whereas 15 patients received image-guided radiation therapy, and one patient received a combination of BT and EBRT. The estimated mean survival was 65.6 months (about five and a half years), with the recurrence-free survival being 61.7 months (about five years). Disease recurrence was diagnosed after radiological or clinical evidence, and recurrence at vaginal stump was subjected to biopsy and histopathological evaluation. In this study cohort, seven patients died during follow-up. Among the patients with lymph node-positive disease, only one was found to have disease recurrence despite adjuvant treatment (Table 2).

**Table 2:** Outcomes post-pelvic lymphadenectomy in early-stage endometrial cancer

Outcomes after pelvic lymphadenectomy		
Type of surgery	Open	34.8%
	Laparoscopic	65.2%
Radiological lymphadenopathy	Yes	19.1%
	No	80.9%
Adjuvant chemotherapy	Yes	18%
	No	82%
Adjuvant radiation	Yes	51.7%
	No	48.3%
Lymph nodes	Positive	6.7%
	Negative	93.3%

**Figure 1:**
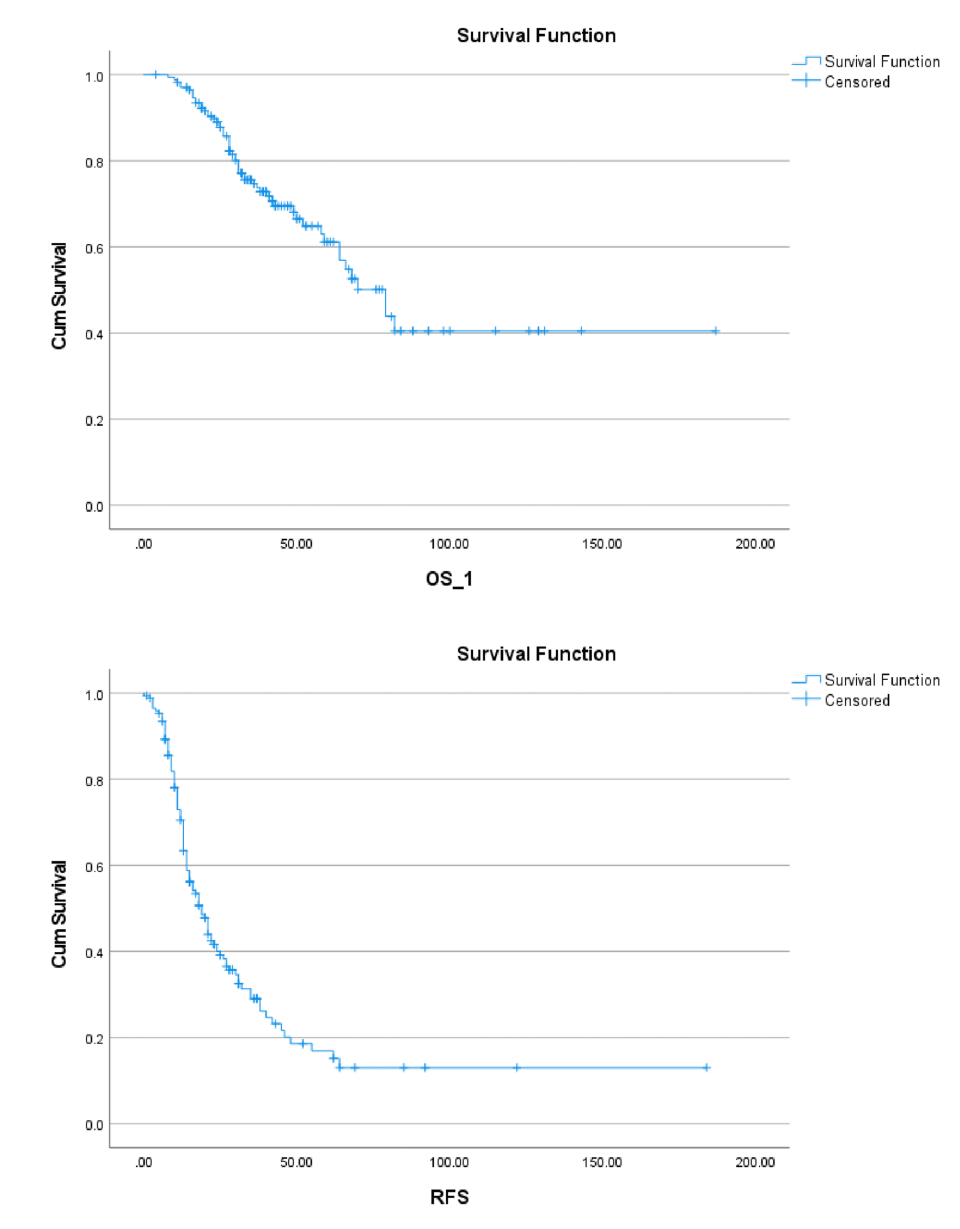
Kaplan-Meier survival curves OS_1: overall survival; RFS: recurrence-free survival

The Kaplan-Meier survival curves for this study cohort illustrating progression-free and overall survival can be seen in Figure 1. 

## Discussion

Surgical staging for early-stage endometrial cancer has been the standard of care before the advent of sentinel lymph node mapping [8]. While the usefulness of sentinel lymph node mapping is being accepted worldwide, centers that do not have this facility are still faced with the conundrum regarding when to do systemic lymph node dissection in view of its subsequent morbidity and effect on progression-free and overall survival [9].

The limitations of this study include its retrospective design with potential for selection bias and missing data. Only six patients had nodal-positive disease; therefore, this sample size might be underpowered to detect statistically significant differences in survival outcomes. Also, since the study is from a single institution and focused on a specific population, findings may not be generalizable to all healthcare settings.

This study has several strengths, including a comprehensive review of patient data over a five-year period and its relevance to clinical practice in low-resource settings where advanced techniques like sentinel lymph node mapping are not readily available. 

In 2020, Zhang et al. conducted a retrospective analysis on 36 patients who were determined to have a very low risk of lymph node metastasis with the help of radiological imaging including a positron emission tomography (PET)-magnetic resonance imaging (MRI) scan preoperatively. Surgical approach included robotic surgery, vaginal hysterectomy, and laparoscopic-assisted vaginal hysterectomy. About 22.2% of patients were found to have high-grade histology on the final specimen. All patients had pelvic and para-aortic lymph node dissection; cytology was taken as decided by the operating surgeon. None of them were found to be positive for lymph node metastasis; therefore, it was concluded that patients who had a low chance of having lymph node metastasis upon initial evaluation with imaging, including PET-MRI, could safely skip surgical lymph node sampling [5].

Otsuka et al. conducted a study that evaluated the role of open surgery including lymphadenectomy without adjuvant therapy highlighting the impact of lymph node dissection on survival and recurrence outcomes. As this study did not include adjuvant therapy, it can be argued that the outcomes are to be attributed to surgery alone. They concluded that when performing an open surgery for endometrial cancer including lymphadenectomy, it can potentially exclude the need for adjuvant therapy. This conclusion indicates the possible therapeutic role of lymph node dissection in endometrial cancer management [10].

Zhai et al. reviewed sentinel lymph node mapping in endometrial cancer, comparing it with systemic lymphadenectomy and selective lymphadenectomy (using Mayo's criteria). They concluded that studies done to assess the role of systemic lymphadenectomy in endometrial cancer were not able to prove any survival benefit and even with selective lymphadenectomy, due to the high sensitivity and low specificity of patient selection criteria, 80% of patients underwent excessive invasive surgery with lymph node dissection. On the other hand, sentinel lymph node biopsy can address this by providing information about lymph node status directing adjuvant therapy while minimizing the morbidity associated with pelvic lymph node dissection [11]. 

Another interesting perspective is provided by a recent study conducted by Bollino et al. in 2024 about lymph node sampling in case of failed sentinel lymph node mapping. According to the European Society of Gynaecological Oncology (ESGO) guidelines, a full side-specific pelvic lymphadenectomy is recommended in case of failed sentinel lymph node mapping [12]; this study discusses an alternative to this by removing selective lymph nodes at the proximal obturator and interiliac positions as identified as the typical sites of metastatic sentinel lymph nodes. The obturator fossa was the predominant location for nodal metastasis [13]. These findings can be extrapolated and possibly considered in low-resource centers without sentinel lymph node mapping. 

A review by Obermair and Abu-Rustum published in 2020 talked about the role of sentinel lymph node mapping in endometrial cancer. Sentinel lymph node mapping entails injecting indocyanine green (ICG) into the cervix and using near-infrared technology to detect the sentinel lymph node during surgery. The aim of this technique is to minimize the number of lymph node dissections where feasible while not compromising on adequate surgical staging and its role in determining adjuvant therapy for patients. While this review raised the important questions of standardizing the technique of lymph node mapping, issues with ultrastaging, and impact on patient outcomes, it is important to note that it mentions a global consensus regarding the usefulness of the sentinel lymph node mapping technique as standard of care in the surgical management of endometrial cancer [14].

The review of previous and ongoing studies clarifies that while sentinel lymph node is gradually becoming the standard practice in endometrial cancer surgery, low-resource centers that do not have this facility still require efficient and accurate preoperative determination of whether a patient should have lymph node dissection to stage the disease. In our study, we have tried to address this by reviewing patients with early endometrial cancer who underwent pelvic lymph node dissection and were found to have lymph node metastasis. Our results show that while lymph node dissection was carried out when indicated preoperatively in a majority of this study cohort, some patients had no high-risk features that could have prompted surgical planning for lymph node dissection performed at the discretion of the operating surgeon. This illustrates that until sentinel lymph node mapping is available, pelvic lymph node dissection remains an important part of surgical staging in endometrial cancer. Selective lymph node dissection in terms of patient selection and anatomical selection of site and extent of lymph node dissection can be up for debate as seen in the studies mentioned above. 

## Conclusions

This study elucidates that patients who underwent pelvic lymphadenectomy and were subsequently found to have nodal disease went on to receive adequate adjuvant therapy; however, for some of them, there was no specific preoperative indicator to prompt the decision for pelvic lymphadenectomy. It can be concluded that pelvic lymphadenectomy in early-stage endometrial cancer facilitates the identification of nodal disease, enabling the administration of appropriate adjuvant therapy. However, given the small number of nodal-positive cases and the retrospective design, these findings should be interpreted with caution. While preoperative imaging may miss nodal involvement, surgical staging remains valuable, particularly in low-resource settings where sentinel lymph node mapping is not yet available. Therefore, until advanced techniques such as sentinel lymph node mapping are available in low-resource countries, surgical staging with pelvic nodal sampling is recommended.
